# The Effect of Closed Incision Negative Pressure Wound Therapy for Prophylactic Use of Postoperative Deep Sternal Wound Infection in Patients Following Coronary Artery Bypass Grafting: A Single-Institutional Study

**DOI:** 10.5761/atcs.oa.26-00048

**Published:** 2026-06-13

**Authors:** Koji Tsutsumi, Nozomi Yamanaka, Misato Tokioka, Osamu Ishida

**Affiliations:** Department of Cardiovascular Surgery, National Defense Medical College, Tokorozawa, Saitama, Japan

**Keywords:** negative pressure wound therapy, sternal wound infection, prevention

## Abstract

**Purpose:**

The aim of this study was to evaluate the effectiveness of closed-incision negative pressure wound therapy (ciNPWT) as a prophylactic measure to prevent deep sternal wound infection (DSWI) after surgery.

**Methods:**

A total of 209 patients undergoing isolated coronary artery bypass grafting via median sternotomy were enrolled. The patients were divided into 3 groups according to incisional care. In Group A (n = 63), the wound was covered with sterile gauze dressings alone. In Group B (n = 71), an indwelling subcutaneous drain was placed and the wound was covered with a hydrocolloid dressing. In Group C (n = 75), an indwelling subcutaneous drain was placed and the wound was covered with a ciNPWT device (PICO; Smith & Nephew). The incidence of DSWI occurring within 60 postoperative days was compared among the 3 groups.

**Results:**

There were no significant differences in preoperative, operative, or postoperative variables among the groups. Postoperative DSWI occurred in 11.1% (7/63) of patients in Group A and 5.6% (4/71) of patients in Group B, whereas no patients in Group C developed DSWI.

**Conclusions:**

Immediate application of NPWT was associated with a reduced rate of wound infection. This treatment may represent an effective preventive strategy.

## Introduction

**Fig. 1:**
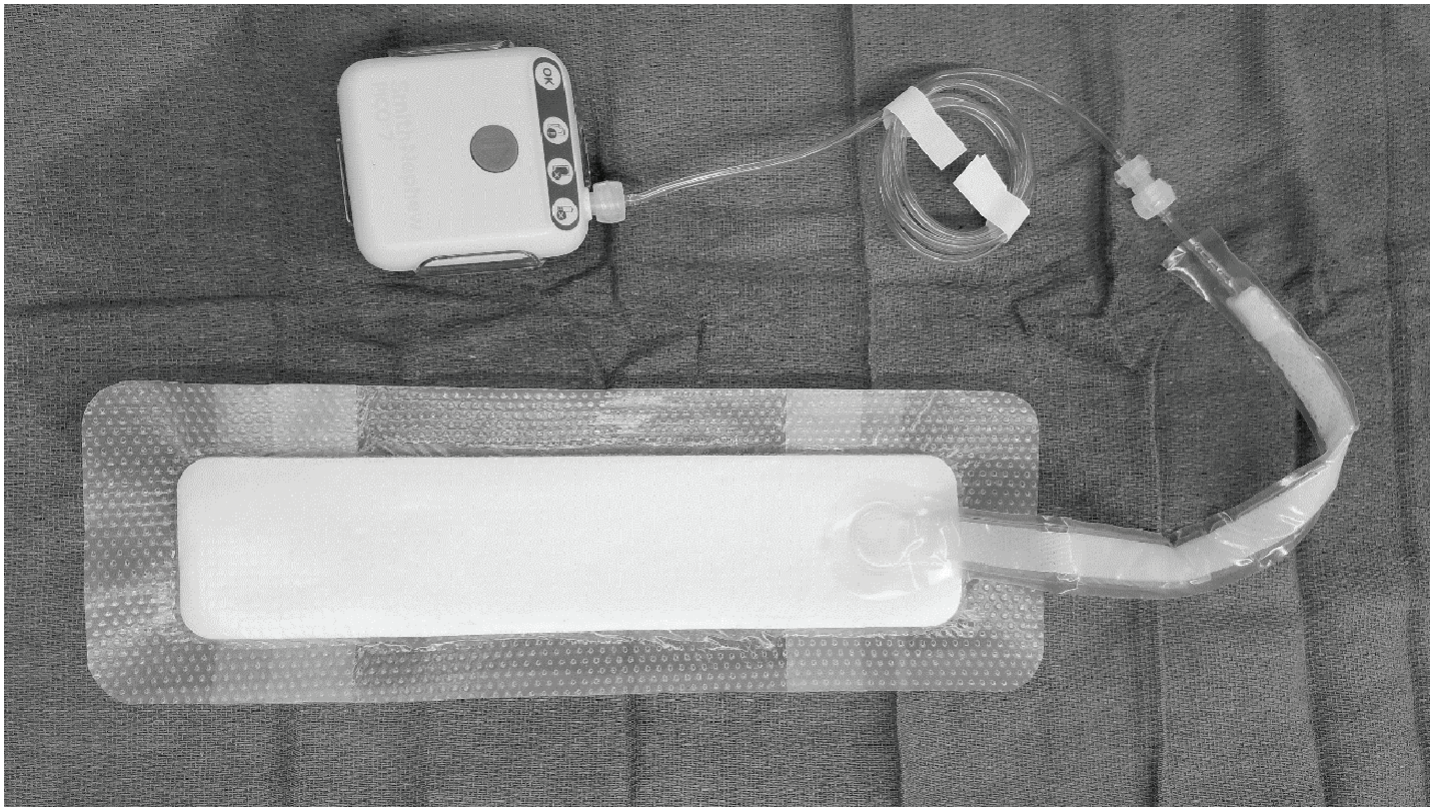
Photograph of the closed-incision management system used for negative pressure wound therapy in this study (PICO 7 Incision Management System; Smith & Nephew Inc.).

Median sternotomy has long been employed as the standard approach for open-heart surgery. However, deep sternal wound infection (DSWI) remains a major complication of cardiac surgery using this approach, with an incidence rate of 0.3%–10.0%.^1–5)^ DSWI is not only associated with increased in-hospital mortality but also adversely affects mid- and long-term survival, resulting in a significant reduction in quality of life and increased healthcare costs. Moreover, DSWI increases the risk of obstruction of internal mammary artery (IMA) grafts.^4,5)^ Although several risk factors have been suggested, such as poorly controlled diabetes mellitus, obesity, or harvesting of bilateral IMAs, the precise pathogenetic mechanisms underlying postoperative DSWI remain unclear.^1–5)^ Although there are many treatments available for DSWI, evidence-based guidelines for the treatment of postoperative DSWI have not been established.^1–3)^ Given the current circumstances, prophylactic management is regarded as the most effective and important strategy. Various techniques and devices have been reported to prevent DSWI, such as prophylactic systemic antibiotics, rigid-plate sternal fixation, or application of hydrocolloid dressings.^1–3)^ Unfortunately, none has achieved widespread use because of the absence of strong evidence. Closed-incision negative pressure wound therapy (ciNPWT) is a concept initially introduced to assist in the treatment of otherwise incurable open wounds due to trauma or infection.^6)^ Recently, there has been growing interest in using the technique on primarily closed surgical incisions to prevent the development of DSWI after cardiovascular surgery.^7–9)^ The PICO 7 Incision Management System (PICO; Smith & Nephew Inc., Watford, UK) is a commercially available ciNPWT system (**Fig. 1**). Since March 2021, the PICO system has been routinely used as a prophylactic measure against DSWI in all patients undergoing cardiac surgery via median sternotomy at Japan’s National Defense Medical College. This study aimed to retrospectively evaluate the efficacy of this approach in preventing DSWI using a non-randomized design.

## Material and Methods

### Patients and the change in postoperative surgical wound management

**Fig. 2:**
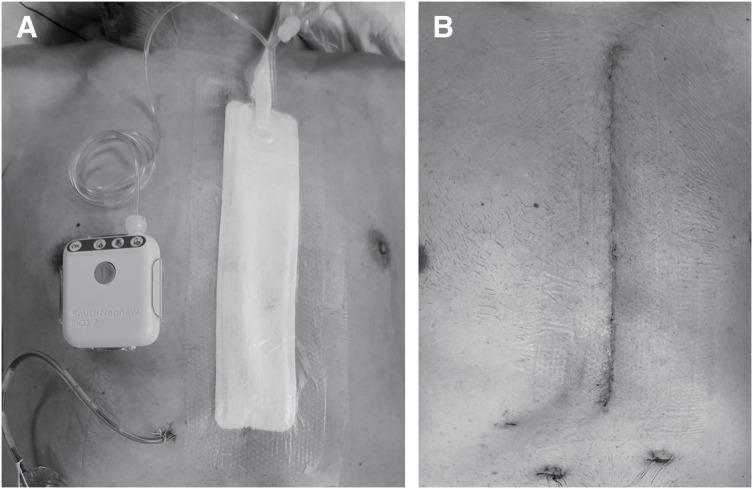
(**A**) The PICO system was applied intraoperatively to the surgical incision after primary wound closure. (**B**) Surgical incision immediately after PICO system removal on postoperative day 7. Complete wound healing was achieved.

A retrospective, single-center cohort study was conducted to evaluate a stepwise implementation of preventive strategies for DSWI after coronary artery bypass grafting (CABG). The Institutional Review Board (IRB) at the National Defense Medical College approved this study (IRB: No. 5050) and waived the requirement for individual consent because of its retrospective nature. The study population consisted of 209 patients who underwent isolated CABG between July 2015 and September 2025. Patients with any of the following conditions were excluded from the study: (1) requirement for harvesting bilateral IMA—bilateral IMA grafting was not routinely performed at our institution during the present study period; (2) need for additional surgical procedures—such procedures could affect both the total operative time and the aortic cross-clamp time; and (3) presence of skin disease or preoperative signs of inflammation—these factors were considered to affect the wound healing process. The surgical site was monitored until postoperative day 60. With the exception of sternotomy incisional care, all operative procedures and perioperative protocols, including infection prevention measures and blood glucose management during the first 7 days after the operation, remained unchanged throughout the study period. According to the postoperative management strategies applied for sternotomy incisional care, the 209 patients were categorized into 3 groups. The sternotomy incisional care within each group was managed in accordance with a uniform, standardized protocol. In Group A (n = 63; July 2015 to September 2019), the incision was covered with conventional sterile gauze dressing only, which was changed every other day until postoperative day 7; thereafter, the wound was left open. In Group B (n = 71; October 2019 to February 2021), an indwelling subcutaneous drain (Blake 15 French size; Johnson & Johnson Inc., New Brunswick, NJ, USA) was placed at the wound site and removed on postoperative day 2. The incision was covered intraoperatively with a transparent hydrocolloid dressing, which was removed on postoperative day 7; thereafter, the wound was left open. In Group C (n = 75; March 2021 to September 2025), in addition to an indwelling subcutaneous drain, the surgical incision was covered intraoperatively with a PICO device (**Fig. 2A**). The PICO system was applied to all patients in Group C. The PICO system remained in place until postoperative day 7, after which it was removed and the wound was left open (**Fig. 2B**). Preoperative, operative, and postoperative variables associated with the development of DSWI were retrospectively collected from medical records. Preoperative variables were collected within 1 month prior to admission. The history of smoking was defined as cessation of smoking at any time prior to the admission, whereas current smoking was defined as the presence of a smoking habit at the time of admission. Morbid obesity was defined as body mass index (BMI) >30 kg/m^2^; poorly controlled diabetes mellitus was defined as hemoglobin A1c (HbA1c) level >8.0%; chronic kidney disease was defined as serum creatinine level >1.5 mg/dL. Postoperative hyperglycemia was defined as a mean blood glucose level >200 mg/dL during the first 7 days after surgery.

### Properties of the ciNPWT device used at the National Defense Medical College

The PICO system is a single-use, canister-free device that uses a battery-powered pump to generate an effective negative pressure of −80 mmHg and is connected to a dressing that removes fluid from the wound site through a unique combination of absorbency and evaporation. This PICO system is so small that patients can be allowed early postoperative ambulation with the PICO still in place.

### Insurance coverage criteria for ciNPWT

Currently, under the Japanese healthcare insurance system, this procedure is not reimbursed or billable as a standalone intervention when its sole purpose is to reduce the risk of DSWI in patients undergoing cardiovascular surgery via median sternotomy. However, insurance coverage may be granted for patients considered at high risk of developing DSWI postoperatively when the procedure is indicated for the promotion of wound healing or the treatment of intractable wounds. Coverage may be approved in cases in which the Special Intensive Care Unit Management Fees are claimed and the following additional conditions are met: (a) obesity, BMI >30; (b) diabetes mellitus, an HbA1c >7.0% based on the National Glycohemoglobin Standardization Program (NGSP) reference values; (c) currently taking steroids or immunosuppressive drugs; (d) chronic hemodialysis; (e) immunocompromised state; (f) malnutrition; (g) skin disorders in which impaired wound healing is anticipated, and (h) patients undergoing redo sternotomy. If a patient does not meet the above criteria, the procedure cannot be billed separately and its cost is covered within the surgical procedure fee.

### Preoperative preparation, surgical practice, and postoperative management at the National Defense Medical College

Patients were showered and shaved the day before the operation. Patients received intravenous cefazolin immediately prior to surgery, followed by repeat boluses every 8 h until postoperative day 2. The operative field was sterilized with povidone–iodine solution, and the skin was covered with an iodine compound–impregnated adhesive plastic sheet. The sternum was divided using a bone saw, and bone wax was routinely used for sternal hemostasis. All single IMAs (predominantly the left) were harvested as in situ pedicled grafts, which were anastomosed to the left anterior descending artery. Other target coronaries were bypassed with saphenous vein grafts. CABG was performed under cardiopulmonary bypass with or without cardiac arrest. Off-pump CABG was not performed during the study period. Prior to sternal closure, the operative field was washed with 3000 mL of normal saline. Sternal re-approximation was performed with 8 separate stainless-steel wires. After closing the sternum, the wound site was also washed with an additional 500 mL of normal saline. The presternal space was obliterated with 4-layer running absorbable sutures. Beginning in October 2019, a subcutaneous drain was also placed on the sternum. The blood glucose levels were monitored 4 times daily until postoperative day 7, and insulin was administered as needed to control blood glucose levels.

### Definition of DSWI and endpoints

In the present study, DSWI was clinically diagnosed within 60 days after surgery when at least one of the following findings was present: (1) purulent discharge from the sternal wound; (2) a positive bacterial culture from the mediastinum or wound; (3) chest pain or tenderness, sternal instability, fever (>38°C), or leukocytosis; and (4) a fluid collection, abscess, or air beneath the sternum detected by computed tomography. Because DSWI is not classified as a standalone category in the Centers for Disease Control and Prevention/National Healthcare Safety Network (CDC/NHSN) “Surgical Site Infection Event” surveillance definitions, DSWI was diagnosed by Koji Tsutsumi according to the previously described criteria. Cases were subsequently categorized within the CDC/NHSN surgical site infection framework based on the deepest level of tissue involvement following median sternotomy, and severity was assessed using clinical and imaging findings. The primary endpoint of the study was the occurrence of DSWI within the 60-day postoperative monitoring period. The secondary endpoint was the development of wound complications, such as superficial wound dehiscence, which did not require extensive debridement or drainage. The diagnostic criteria and surveillance protocol for DSWI remained consistent throughout the study period.

### Statistical analysis

Continuous data were reported as mean ± standard deviation or median (interquartile range), and categorical data were reported as numbers (%). Comparisons between the 3 groups were performed using 1-way analysis of variance. Because DSWI events were rare and no events occurred in Group C, standard logistic regression was susceptible to separation and small-sample bias; therefore, we used Firth’s penalized logistic regression to obtain finite, bias-reduced estimates. The preventive strategy was modeled as an ordered exposure (Group A to Group C). The primary model evaluated the association between groups and DSWI adjusted for prespecified clinical covariates (BMI and HbA1c), and results are presented as adjusted odds ratios (aORs) with 95% confidence intervals (CIs) derived from the profile penalized likelihood. Sensitivity analyses were conducted by additionally adjusting for intraaortic balloon pumping (IABP) use and the on-pump beating technique in separate models. Statistical analyses were performed using EZR (an R-based statistical interface; Saitama Medical Center, Jichi Medical University, Saitama, Japan)^10)^ and R (version 4.5.2; The R Foundation for Statistical Computing, Vienna, Austria), with Firth’s penalized logistic regression implemented via the logistf package (version 1.26.1). A 2-sided p value <0.05 was considered statistically significant.

## Results

### Preoperative risk factor analysis

Preoperative patient characteristics are summarized in **Table 1**. Overall, demographic and comorbidity profiles were broadly comparable across groups, with some differences consistent with real-world case mix; for example, the incidence of acute myocardial infarction (6.3%, 2.8%, and 12.0%) and the proportion of patients requiring IABP (38.1%, 25.4%, and 14.7%) differed among the groups. There were no significant differences in the prevalence of other preoperative variables, such as age, BMI, severity of diabetes mellitus, preoperative cardiac function, EuroSCORE II, chronic renal failure requiring regular hemodialysis, or the rate of emergent operation between the 3 groups.

**Table 1:** Preoperative patient’s characteristics

Preoperative data	Group A (63)	Group B (71)	Group C (75)	*p* Value
Age (years)	70.0 ± 10.3	69.0 ± 12.0	69.4 ± 10.7	0.87
Male	51 (81.0)	55 (77.5)	51 (68.0)	0.52
BMI	23.1 ± 4.0	23.4 ± 3.7	24.0 ± 5.3	0.57
Obesity (BMI >25.0)	13 (20.6)	20 (28.2)	19 (25.3)	0.53
Unstable angina pectoris	18 (28.6)	21 (29.6)	18 (24.0)	0.98
Acute myocardial infarction	4 (6.3)	2 (2.8)	9 (12.0)	0.03
Emergent operation	14 (22.2)	12 (16.9)	9 (12.0)	0.38
Cancer	14 (22.2)	9 (12.7)	8 (10.7)	0.30
History of smoking	33 (52.4)	38 (53.5)	41 (54.7)	0.98
Current smoking	9 (14.3)	14 (19.7)	9 (12.0)	0.43
Serum albumin level	3.6 [3.1, 4.0]	3.8 [3.5, 4.2]	3.8 [3.4, 4.1]	0.06
Liver cirrhosis	5 (7.9)	2 (2.8)	2 (2.7)	0.39
COPD	5 (7.9)	7 (9.9)	3 (4.0)	0.16
Steroids use	0 (0)	4 (5.6)	1 (1.3)	0.14
Serum creatinine (mg/dL)	1.0 [0.8, 2.8]	1.0 [0.7, 1.4]	1.1 [0.8 1.4]	0.22
Preoperative CKD (Cr >1.5)	15 (23.8)	13 (18.3)	16 (21.3)	0.52
Preoperative regular HD	11 (17.5)	10 (14.1)	12 (16.0)	0.39
Diabetes mellitus	25 (39.7)	31 (43.7)	29 (38.7)	0.19
HbA1c (%)	6.0 [5.6, 6.9]	6.1 [5.7, 7.0]	6.5 [6.1, 6.9]	0.14
Brain natriuretic peptide (pg/mL)	75.2 [44.4, 416.2]	68.7 [20.9, 159.3]	87.4 [22.0, 217.2]	0.15
Left ventricular ejection fraction (%)	51.7 ± 15.2	54.7 ± 13.8	50.2 ± 14.1	0.21
Depression of left ventricular function	15 (23.8)	11 (15.5)	13 (17.3)	0.42
IABP required	24 (38.1)	18 (25.4)	11 (14.7)	0.03
EuroSCORE II	3.0 [1.8, 5.3]	3.1 [2.1, 5.9]	2.2 [1.3, 4.5]	0.78
Redo sternotomy	1 (1.6)	0 (0)	0 (0)	0.63

Continuous data are reported mean ± standard deviation (SD) or median and interquartile range [IQR]. Categorical data are reported as number (%).

BMI, body mass index; COPD, chronic obstructive pulmonary disease; CKD, chronic kidney disease; HD, hemodialysis; HbA1c, hemoglobin A1c; IABP, intraaortic balloon pumping; Cr, creatinine

### Operative and postoperative risk factor analysis

Operative and postoperative variables are shown in **Table 2**. The frequency of on-pump beating CABG was lower in Group C (8.0%) than in Groups A and B (31.7% and 31.0%, respectively), and cardiopulmonary bypass and cross-clamp times were significantly longer in Group C. In Group C, a greater number of total bypass sites were observed as compared to Groups A and B. The mean postoperative blood glucose level during the first 7 postoperative days tended to be higher in Group C than in the other 2 groups. However, there were no significant differences in other operative and postoperative variables, such as operation time, volume of bleeding, intubation time, or re-exploration rate between the 3 groups.

**Table 2:** Operative and postoperative patient’s characteristics

	Group A (63)	Group B (71)	Group C (75)	*p* Value
Operative data				
Operation time (min)	358 [314, 395]	355 [323, 397]	375 [336, 408]	0.41
CPB time (min)	121 [98, 144]	131 [114, 151]	140 [123, 158]	0.02
Aorta clamp time (min)	65 [0, 81]	64 [0, 86]	81 [67, 101]	0.00
On-pump beating CABG	20 (31.7)	22 (31.0)	6 (8.0)	0.00
LITA use	60 (95.2)	68 (95.8)	73 (97.3)	0.53
RITA use	3 (4.8)	3 (4.2)	2 (2.7)	1.00
Number of bypass grafts	2 [1, 2]	2 [1, 2]	2 [2, 3]	0.00
Total blood loss (mL)	483 [357, 614]	603 [456, 729]	552 [407, 670]	0.07
Re-exploration	1 (1.6)	2 (2.8)	1 (1.3)	1.00
Postoperative data				
Subcutaneous drainage output (mL)	NA	13 [16, 10]	14 [9, 18]	0.25
Mean BG (mg/dL)	169 [148, 189]	159 [144, 185]	183 [154, 201]	0.05
Max creatinine (mg/dL)	1.3 [1.1, 3.3]	1.3 [1.0, 2.1]	1.3 [1.0, 1.9]	0.29
Ventilation time (h)	16 [14, 17]	15 [14, 16]	16 [14, 17]	0.54
ICU stay (day)	3 [2, 3]	2 [2, 3]	2 [2, 3]	0.00
Re-exploration	1 (1.6)	2 (2.8)	1 (1.3)	0.79
CHDF	19 (30.2)	19 (26.8)	17 (22.7)	0.81
Hospital stay (day)	20 [16, 30.5]	19 [15, 23.5]	21 [16, 29]	0.21
DSWI	7 (11.1)	4(5.6)	0 (0)	0.03
Superficial wound dehiscence	1 (1.6)	2 (2.8)	0 (0)	1.00
In-hospital mortality	1 (1.6)	0 (0)	0 (0)	1.00

CPB, cardiopulmonary bypass; CABG, coronary artery bypass surgery; LITA, left internal thoracic artery; RITA, right internal thoracic artery; NA, not applicable; mean BG, mean blood glucose level during the first 7 postoperative days; ICU, intensive care unit; CHDF, continuous hemodialysis and filtration; DSWI, deep sternal wound infection

### Incidence of DSWI and length of hospital stay decreased with the use of the PICO system

A total of 11 patients developed DSWI: 7/63 (11.1%) in Group A, 4/71 (5.6%) in Group B, and 0/75 (0%) in Group C (**Table 3a**). The characteristics of the patients who developed DSWI are shown in **Table 4**. The mean interval from surgery to the onset of DSWI was 18.4 days. In the present study population, no patients developed DSWI beyond postoperative day 60. The mean hospital length of stay after developing DSWI was 143.2 days. In all patients with DSWI, the infected wound sites were opened and surgically debrided. Following this procedure, 5 patients were treated with only ciNPWT and the remaining 6 patients were treated with ciNPWT and delayed closure with omental flap transposition. One patient out of the 11 patients died, resulting in a mortality rate of 9.1%. There were no in-hospital deaths in the other 198 patients who did not develop postoperative mediastinitis. Superficial wound dehiscence under aseptic conditions was observed in 1 patient from Group A (1.6%), 2 patients in Group B (2.8%), and no patients in Group C (0%). All 3 wound dehiscences healed completely without requiring extensive debridement or drainage.

**Table 3:** (a) DSWI case counts within each group and (b) summary of regression analysis of DSWI

(a) DSWI case counts within each group					
			Group A (63)	Group B (71)	Group C (75)
DSWI (−)			56 (88.9)	67 (94.4)	75 (100)
DSWI (+)			7 (11.1)	4 (5.6)	0 (0)
(b) Summary of regression analysis of DSWI					
Model	Outcome	Exposure	Covariates	Statistical method	Effect estimate
Primary model	DSWI	Preventive strategy modeled as an ordered exposure (Group A–B–C)	BMI, HbA1c	Firth’s penalized logistic regression	aOR per 1-stage increase: 0.31 (95% CI 0.10–0.75; p = 0.01)
Sensitivity Model 1	DSWI	Preventive strategy modeled as an ordered exposure (Group A–B–C)	BMI, HbA1c, IABP use	Firth’s penalized logistic regression	aOR per 1-stage increase: 0.33 (95% CI 0.11–0.81)
Sensitivity Model 2	DSWI	Preventive strategy modeled as an ordered exposure (Group A–B–-C)	BMI, HbA1c, on-pump beating CABG	Firth’s penalized logistic regression	aOR per 1-stage increase: 0.31 (95% CI 0.10–0.75)

Preventive strategy was analyzed as an ordered exposure reflecting stepwise intensification of postoperative incisional care from Group A to Group C.

aOR, adjusted odds ratio; CI, confidence interval; DSWI, deep sternal wound infection; BMI, body mass index; HbA1c, hemoglobin A1c; CABG, coronary artery bypass surgery

**Table 4:** Characteristics of the patients who developed DSWI after surgery

Preoperative risk factor	Days to onset	In-hospital period	Pathogen	Treatment	Outcome
HD	14	67	MSSA	ciNPWT	Alive
Cancer carrier	14	101	Serratia M	ciNPWT	Alive
HD	13	139	MSSA	ciNPWT	Alive
DM	9	195	*E. coli*	ciNPWT	Alive
HD, obesity, DM	15	138	MRSA	ciNPWT+TFT	Alive
Cardiogenic shock due to AMI, renal failure	15	27	*S. epidermidis*	ciNPWT	Dead
DM, HD, obesity	29	64	MSSA	ciNPWT+TFT	Alive
Obesity, DM	21	195	MSSA	ciNPWT+TFT	Alive
HD, obesity, DM	12	315	*S. epidermidis*	ciNPWT+TFT	Alive
HD, DM	28	92	MRSA	ciNPWT+TFT	Alive
HD, DM	15	242	MRSA	ciNPWT+TFT	Alive
Mean (day)	18.4	143.2			

HD, hemodialysis; DM, diabetes mellitus; AMI, acute myocardial infarction; MSSA, Methicillin-sensitive *Staphylococcus aureus*; MRSA, Methicillin-resistant *Staphylococcus aureus*; *S. epidermidis*, *Staphylococcus epidermidis*; Serratia M, *Serratia marcescens*; *E. coli*, *Escherichia coli*; ciNPWT, closed incision negative pressure wound therapy; TFT, tissue flap transposition

### Bacterial pathogens identified in infected wound sites

Methicillin-sensitive *Staphylococcus aureus* was isolated from 4 wound sites (36.4%) and Methicillin-resistant *Staphylococcus aureus* from another 3 sites (**Table 4**). In the remaining wounds with signs of microbial infection, several other bacterial species were isolated.

### Possible preoperative factors for patients predisposed to developing DSWI

Among the 11 total patients who developed DSWI, we identified several potential risk factors that may have contributed to the development of postoperative DSWI (**Table 4**). These factors included preoperative hemodialysis (7/11 patients), poorly controlled diabetes mellitus (7/11 patients), morbid obesity (4/11 patients), acute myocardial infarction with cardiogenic shock (1/11 patients), and postoperative hyperglycemia (2/11 patients).

### Statistical analysis

Results of statistical analyses are shown in **Table 3b**. In the primary Firth’s penalized logistic regression model adjusted for BMI and HbA1c, a stepwise improvement in the preventive strategy for post-sternotomy incisional care was associated with significantly lower odds of DSWI, with an adjusted odds ratio per 1-stage increase of 0.31 (95% CI: 0.10–0.75; p = 0.01). BMI (aORs: 1.08, 95% CI: 0.93–1.22; p = 0.29) and HbA1c (aORs: 1.14, 95% CI: 0.62–1.85; p = 0.65) were not independently associated with DSWI in this model. The association between the stepwise preventive strategy for post-sternotomy incisional care and DSWI remained consistent in sensitivity analyses that additionally adjusted for IABP use (stage aORs: 0.33, 95% CI: 0.11–0.81) and for on-pump beating CABG (stage aORs: 0.31, 95% CI: 0.10–0.75).

## Discussion

### Primary findings of the present study

In this single-center cohort of 209 patients undergoing isolated CABG, we observed a stepwise reduction in the incidence of DSWI associated with progressive intensification of preventive strategy for post-sternotomy incisional care, culminating in zero DSWI events following the introduction of ciNPWT in addition to subcutaneous drainage. Importantly, there were no significant differences among the 3 groups in baseline preoperative, operative, or postoperative variables known to influence the incidence of DSWI. Using Firth’s penalized logistic regression, which is appropriate for rare events and complete separation, we found that each 1-stage intensification of the preventive strategy was associated with a substantial reduction in the odds of DSWI (aOR approximately 0.31). This association remained stable after additional adjustment for IABP use or the on-pump beating technique. These findings suggest that the implementation of ciNPWT as part of a stepwise preventive strategy may independently contribute to reducing the risk of DSWI following CABG.

### Clinical background and risk factors for DSWI

Median sternotomy wound healing is influenced by multiple patient- and surgery-related factors. Patient-related factors include morbid obesity, poorly controlled diabetes mellitus, steroid use, preoperative malnutrition, chronic renal failure, and CABG itself, particularly when bilateral IMAs are harvested.^1–5)^ Surgery-related factors include surgical site contamination, suture technique, cardiopulmonary bypass time, and overall operative time.^1–5)^ In the present study, several of these factors, such as the operating surgeon and the method of surgical wound closure, remained constant, thereby minimizing variability in these variables and allowing a clearer assessment of the preventive strategy.

### Potential mechanisms of ciNPWT

Several mechanisms have been proposed to explain the effectiveness of ciNPWT.^7–9)^ (a) The first theory is that the ciNPWT may remove excessive interstitial fluid and toxic inflammatory mediators from the subcutaneous tissue. This effect can decrease cutaneous edema, increase cutaneous blood flow to the wound margins, and promote angiogenesis, which may further stimulate the growth of granulation tissue and sternal stabilization. (b) The second theory is that ciNPWT dressings may mechanically stabilize incision edges together and reduce lateral stresses on the incision margins by approximately 50%, which ends up preventing skin breakdown. This is particularly important for patients with morbid obesity. (c) The third theory suggests that ciNPWT may enhance local immune responses, improve fibroblast viability, and decrease bacterial colony counts. These mechanisms together may contribute to improving wound healing and reducing the risk of infection.

### Implementation of preventive strategies for DSWI at the National Defense Medical College

At the National Defense Medical College, DSWI was most frequently observed in patients undergoing CABG, consistent with previous reports identifying CABG itself as a potential risk factor for DSWI,^1–5)^ thereby highlighting the clinical need for preventive strategies. Initially, a subcutaneous drain was placed to reduce subcutaneous fluid accumulation and dead space, thereby maintaining a cleaner wound environment and potentially inhibiting bacterial growth. However, DSWI still occurred, particularly in high-risk patients such as those on preoperative hemodialysis. Based on the established efficacy of ciNPWT in managing open infected wounds, we hypothesized that its prophylactic application of ciNPWT to primarily closed surgical incisions could further reduce infection risk. Based on this concept, we began applying ciNPWT to patients undergoing CABG at the National Defense Medical College in March 2021. Since then, no cases of DSWI have been reported, and surgical wound healing outcomes appeared to improve (**Fig. 2B**). These observations were supported by multivariate analysis, reinforcing the potential benefit of ciNPWT as part of a bundled preventive strategy. While standardized indications for prophylactic ciNPWT in clean, closed surgical incisions have not been fully established, our findings suggest that its use may be considered, particularly in patients at elevated risk. However, prevention of DSWI likely requires a multifocal approach, including strict aseptic technique, appropriate antimicrobial prophylaxis, secure sternal fixation, meticulous wound closure, and optimal perioperative glycemic control. ciNPWT may provide the greatest benefit when used in combination with these measures.

### Regulatory and reimbursement considerations in Japan

In the Japanese healthcare system, prophylactic use of ciNPWT is reimbursed under the conditions described above, primarily for patients considered to be at high-risk of wound complications. This clarification is important for interpreting the generalizability of our findings in real-world clinical and regulatory settings.

### Cost considerations and potential health economic implications

Cost is an important consideration, as ciNPWT systems such as PICO are more expensive than conventional dressings.^10)^ The device cost ranges from approximately 30000 to 40000 yen depending on pad size, while reimbursement under the Japanese public health insurance system is typically 20000 to 30000 yen under the aforementioned conditions. In contrast, the mean total medical cost for patients who developed DSWI was approximately 10350000 yen, compared with 3720000 yen for patients without DSWI, representing an approximately 2.8-fold increase. The mean length of hospital stay for patients with DSWI was 150.3 days, whereas it was 20.7 days for those without DSWI, corresponding to an approximately 6.9-fold increase. These findings suggest that the additional cost of prophylactic ciNPWT may be considered cost-effective from a health economic perspective, particularly in patients at high risk for DSWI.

### Limitations

This study has several limitations. First, it was a single-center, retrospective, non-randomized study with a relatively small sample size. Therefore, it may have been underpowered, and effect estimates may be unstable. Second, the observational design precludes definitive conclusions regarding causality. In addition, based on the present results, we were unable to determine with high confidence the exact pathogenic mechanisms underlying the development of DSWI. Third, as comparisons were made across different time periods, the possibility of chronological bias cannot be excluded. Improvements in general perioperative care over time may also have influenced the outcomes. Larger prospective and randomized studies are warranted to validate these findings and further elucidate the mechanisms involved.

## Conclusions

The present findings suggest that ciNPWT may reduce the incidence of DSWI compared with conventional gauze dressings or indwelling subdermal drains covered by transparent hydrocolloid dressings. Furthermore, our findings suggest that ciNPWT may have a substantial beneficial effect in patients with preoperative risk factors who are considered to have increased risk of developing DSWI postoperatively. The PICO system was well tolerated by patients and can be managed easily by medical staff with minimal additional training. DSWI remains a serious and potentially devastating complication following cardiac surgery via median sternotomy. Therefore, the prophylactic use of ciNPWT may represent a useful option for clinicians to prevent DSWI after cardiac surgery.

## Declarations

### Data availability

The data underlying this article will be shared on reasonable request to the corresponding author and after acceptance by the institutional authority.
